# Coffee and caffeine intake and depression in postpartum women: A cross-sectional study from the National Health and Nutrition Examination Survey 2007–2018

**DOI:** 10.3389/fpsyg.2023.1134522

**Published:** 2023-02-17

**Authors:** Yinuo Wang, Zhuangfu Wang, Peijun Gui, Bo Zhang, Ying Xie

**Affiliations:** ^1^Department of Rehabilitation Medicine, Beijing Friendship Hospital, Capital Medical University, Beijing, China; ^2^UCL Great Ormond Street Institute of Child Health, Faculty of Population Health Science, London, United Kingdom

**Keywords:** depression, coffee, caffeine, postpartum population, postpartum rehabilitation

## Abstract

This cross-sectional study examines the association between coffee and caffeine consumption and depressive symptoms in postpartum women. In total, 821 postpartum women who met the study’s inclusion criteria were interviewed. Data were extracted from the 2007–2018 National Health and Nutrition Examination Survey. Coffee consumption and 11 confounding variables were considered and analyzed as baseline data. Weighted logistic regression models were constructed by adjusting the variables, and the odds ratios of total coffee, caffeinated coffee, and decaffeinated coffee were assessed for their impact on depression status. In addition, subgroup analyses were conducted according to race, breastfeeding status, and postpartum period. The results show that generic coffee and caffeinated coffee intake have a potentially protective effect in postpartum women. Drinking more than three cups of caffeinated coffee may lower the risk of postpartum depression, particularly in the 1–2 year postpartum period and in non-breastfeeding women. The association between decaffeinated coffee consumption and postpartum depression remains unclear.

## Introduction

Postpartum depression (PPD) is one of the most prevalent postpartum complications and affects approximately 8–26% of postpartum women every year (Shorey et al., 2018; Wang and Shan, 2022). Notably, a recent survey has demonstrated that the incidence rate of PPD has increased since the global COVID-19 pandemic (Alsabi et al., 2022; Perez et al., 2021). PPD can hit a woman between 3 and 18 months after giving birth, depending on hormonal changes in the mother’s body (Slomian et al., 2019). Moreover, recent studies have observed that 5% of postpartum women experienced continuously PPD symptoms for even three years after giving delivery (Kolomanska-Bogucka and Mazur-Bialy, 2019; Wan et al., 2021). Meanwhile, PPD is also known to have a long-lasting impact on the mother as well as her newborn and can influence the economic well-being and financial stability of mothers up to 15 years later (Rokicki et al., 2022). The economic burden of PPD is significant and estimated at approximately $14.2 billion annually in the United States (Luca et al., 2019). Therefore, there is an urgent need to investigate protective factors associated with PPD.

Coffee is one of the most popular beverages worldwide, and its primary component, caffeine, is recognized as the most widely used psychoactive substance. According to a survey conducted by the National Coffee Association (NCA), 62% of Americans consume coffee on a daily basis, and the average American coffee drinker consumes more than three cups per day. Generic coffee consumption in the United States has increased by 5% since 2015, prompting academics to conduct more studies on the effects of coffee on generic health (Shehaj and Morina, 2022). Coffee has been shown to lower the risk of cardiovascular disease (Surma et al., 2023), colorectal cancer (Deng et al., 2022), liver cirrhosis (Barre et al., 2022), and other illnesses, but it may also increase the risk of sleep problems (Riera-Sampol et al., 2022), anxiety disorders (Pan et al., 2022), and bone fractures (Webster et al., 2022).

The association between coffee consumption and depression remains debatable. Caffeine can reverse the dopaminergic system impairments observed in depression; for example, inhibiting the A1 adenosine receptor subunit may boost catecholamines and serotonin (5-HT) levels in the central nervous system (CNS) (Fredholm, 1995; Lopez-Cruz et al., 2018), besides, it is also worth noting that Caffeine increases the release of 5-HT in the limbic system and dopamine (DA) in the prefrontal cortex, producing effects similar to those of antidepressants (Acquas et al., 2002; Szopa et al., 2016). A longitudinal study of 50,776 women in the United States observed a negative correlation between coffee consumption and depressive behaviors after adjusting for age. A daily coffee intake of 2–3 cups and 4 cups was related to a 45 and 53% reduction in the risk of suicide, respectively; however, this effect was not observed in the decaffeinated population (Lucas et al., 2011). More recently, Grosso et al. (2016) conducted a meta-analysis that included 23 observational studies. Their results suggested that coffee consumption has a protective effect on the risk of depression, with a peak protective effect of 400 ml/day (Grosso et al., 2016). However, randomized clinical trials on coffee and depression produced contradictory results. In a prospective, randomized crossover trial by Quinlan et al. (2000), tea and coffee were found to exert similar mood and cognitive effects, which could not be explained by the caffeine content and rather the potential effects of other factors, such as flavonoids.

Multiple subpopulations, including college students (Yang et al., 2022), schoolchildren (Richards and Smith, 2015), and older adults (Guo et al., 2014), have been the focus of recent studies exploring the connection between coffee and depression. The data suggest that coffee has a favorable effect on adolescents but a detrimental effect on older adults in terms of depression. Although postpartum women are at an increased risk of depression, there are insufficient data on coffee and postpartum depression. Using the National Health and Nutrition Examination Survey (NHANES) database, we performed a large-scale, nationally representative study between 2007 and 2018 to learn more about the association between coffee consumption and PPD.

The remainder of the paper is structured as follows: Section “Materials and methods” presents the materials and methods used in the study. Section “Results” elaborates on the results of the study. Section “Discussion” presents the findings in the light of previous knowledge as well as the limitations and directions for future research.

## Materials and methods

### Data source and population

This analysis was based on NHANES data from 2007 to 2018. The NHANES is a nationally representative database comprising interviews, examinations, and laboratory data obtained from a multistage, stratified sample of adults and children in the United States, which may reflect the physical and mental well-being status of the total population. The data gathered by the NHANES are accessible to the public and are primarily used by public health researchers, policymakers, and physicians to estimate the prevalence of diseases and risk factors, and to promote the design of appropriate public health policies. The study was authorized by the National Center for Health Statistics Institutional Ethics Review Board, and all participants involved in NHANES study provided written consent.

**FIGURE 1:**
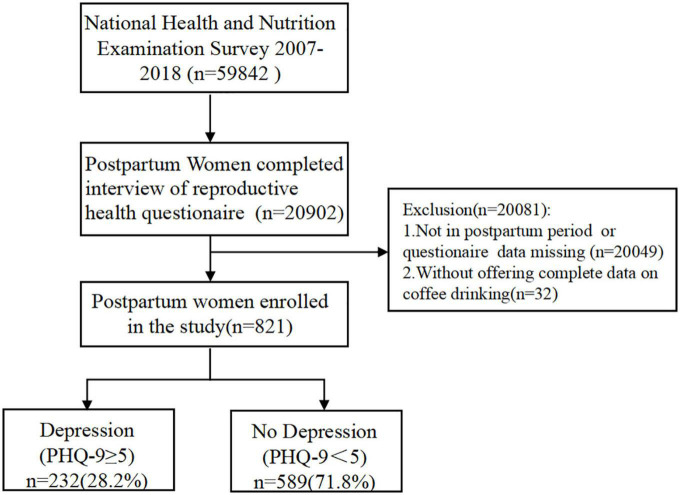
Flowchart illustrating the selection of participants.

A total of 59,842 individuals from six successive NHANES cycles (2007–2008, 2009–2010, 2011–2012, 2013–2014, 2015–2016, and 2017–2018) were selected, of which 20,902 individuals had completed the reproductive health questionnaire. After excluding 20,049 women who were not in postpartum stage or had missing data, as well as two women who did not offer dietary information on their coffee consumption, 821 individuals were included in our investigation. Patient Health Questionnaire-9 items (PHQ-9) scores were simultaneously obtained to evaluate depression. Referring to recent evidence, postpartum depression can still occur up to 2 years postpartum (Kolomanska-Bogucka and Mazur-Bialy, 2019; Wan et al., 2021). Therefore, we included participants several months, 1 year and 2 years after delivery. According to Huyen et al. (2021), a cut-off value of >5 could predict 70% of cases of mild postpartum depression. We used a binarized interval score of 0–4 and more than 5 as the benchmarks for depression diagnosis. Ultimately, 232 cases (28.2%) were classified into the depression group, and 586 cases (71.8%) were classified into the non-depression group (Figure 1). According to the official sampling ratio and weight design, the 821 participants extracted from the NHANES represented 5.87 million noninstitutionalized residents of the United States.

### Coffee and caffeine intake calculation

Two 24-h dietary recall interviews were used to gather data on NHANES participants’ coffee intake. The first dietary recall interview was conducted at a mobile examination center (MEC), while the second interview was conducted one week later via telephone. The NHANES data on coffee consumption included several types of coffee and coffee drinks. This survey detailedly collected the meals and beverages participants consumed in the past day (from midnight that day to midnight the day before) or in the previous 24 h, and synthesizes nutritive information of ingredients according to formula obtained from various sources, including the manufacturer or retailer, Internet or company catalogs. Hence, this method is widely recognized as an effective strategy for evaluating the coffee intake in population (Chen et al., 2022; Geng et al., 2022; Wang et al., 2022; Zheng et al., 2022). Caffeine intake was calculated based on the overall quantity of caffeinated meals and beverages consumed. This study examined coffee, decaffeinated coffee, sugar-sweetened coffee, and total caffeine consumption to analyze the correlation between coffee and its components, and depressive symptoms.

### Covariables

Based on past research on the relationship between coffee and caffeine intake and depression, the following characteristics were added as covariables: age (year), body mass index (BMI, kg/m^2^), and postpartum time (within 1 year/1–2 years/More than 2 years). Race and socioeconomic factors that could influence incidences of PPD were also included: education level (less than high school, high school or equivalent, and above high school), family income-to-poverty ratio (PIR), covered by insurance (yes/no), and marital status (married/living with a partner, widowed/separated/divorced, and never married). In addition, smoking status (never, ever, and currently) and breastfeeding status (yes/no) were included as variables in this investigation.

### Statistical analysis

The MEC sample weight was considered and allocated to each participant in accordance with the NCHS recommendations. Categorical data were presented as frequencies or percentages, while continuous variables were represented as mean ± standard deviation (SD). To quantify the differences between the groups, a weighted analysis of variance (ANOVA) or chi-square test (for categorical variables) was used. In accordance with the guidelines provided by the Reporting on Observational Studies in Strengthening Epidemiology, three weighted logistic regression models were used to calculate the odds ratios (ORs) and 95% confidence intervals (CIs) for PPD in each coffee drinking group. Model 1 was crude and untouched; Model 2 was adjusted for age and race/ethnicity; and Model 3 accounted for all the confounders. In addition, we performed subgroup analyses based on race, postpartum duration, and smoking status to further evaluate the association between coffee intake and PPD. R^1^ and StataSE 15.1 were used for all the statistical analyses (StataCorp LLC, College Station, TX, USA). Statistical significance was set at *p* < 0.05.

## Results

### Description of the study’s baseline characteristics

Table 1 shows the overall weighted characteristics of the study sample. Of the 821 postpartum women, 232 had depressive symptoms, and 589 had no obvious depressive symptoms. There was a significant difference in coffee intake between the depressed and non-depressed groups. The total intake of coffee in the depressed group was 0.73 ± 0.09 cups/day vs. 1.09 ± 0.13 cups/day in the non-depressed group (*p* = 0.02). A statistical difference was also observed for caffeinated coffee consumption: 0.70 ± 0.09 cups per day in the depressed group versus 1.04 ± 0.13 cups per day in the non-depressed group (*p* = 0.03). However, there was no statistically significant difference between the two groups in terms of decaffeinated coffee and sweetened coffee intake. In addition, there were statistical differences in education, household income, poverty rates, breastfeeding status, BMI, and smoking status, although differences in insurance coverage were marginal (*p* = 0.08). No statistically significant differences were observed in terms of race or ethnicity, moderate activity level, or postpartum period. The mean age of the depression group was 28.42 ± 8.62 years old, and the mean age of the non-depressive women was 34.42 ± 8.84 years old, with no statistical significance.

**TABLE 1:** Basic characteristics of the study.

	Total (*n* = 821)	Depression (*n* = 232)	No depression (*n* = 589)	*p*-Value
Age (years)	29.07 ± 0.31	28.42 ± 0.54	29.31 ± 0.37	0.17
Race/ethnicity (%)				0.25
Mexican American	171(20.83)	51(28.54)	120(71.46)	
Non-Hispanic Black	159(19.37)	54(32.40)	105(67.60)	
Non-Hispanic White	297(36.18)	82(26.15)	215(73.85)	
Other	194(23.63)	45(21.90)	149(78.10)	
Educational level, *n* (%)				**0.02**
Above high school	455(55.42)	108(22.17)	347(77.83)	
High school or equivalent	189(23.02)	64(32.72)	125(67.28)	
Less than high school	177(21.56)	60(34.00)	117(66.00)	
Ratio of family income to poverty (PIR)	2.20 ± 0.07	1.90 ± 0.14	2.31 ± 0.08	**0.01**
Marital (%)				
Divorced/ separated/ widowed	40(4.87)	19(42.15)	21(57.85)	**<0.001**
Married/ living with partner	633(77.1)	150(22.88)	483(77.12)	
Never married	148(18.03)	63(41.93)	85(58.07)	
Moderate activity (%)				0.81
Yes	349(42.51)	107(26.13)	242(73.87)	
No	472(57.49)	125(26.94)	347(73.06)	
Breastfeeding (%)				**0.03**
Yes	203(24.73)	45(18.47)	158(81.53)	
No	618(75.27)	187(29.21)	431(70.79)	
Postpartum time (%)				0.13
Within 1 year	130(15.83)	30(20.78)	100(79.22)	
1–2 years	404(49.21)	125(30.22)	279(69.78)	
More than 2 years	287(34.96)	77(23.93)	210(76.07)	
Smoke (%)				**<0.001**
Former	84(10.23)	26(25.59)	58(74.41)	
Never	584(71.13)	138(22.00)	446(78.00)	
Now	153(18.64)	68(42.79)	85(57.21)	
Insurance (%)				0.08
Yes	604(73.57)	160(24.90)	444(75.10)	
No	217(26.43)	72(32.73)	145(67.27)	
BMI (kg/m^2^)	29.54 (28.77,30.31)	31.24 (29.89,32.60)	28.92 (28.08,29.76)	**0.003**
Generic coffee intake (cup/day)	0.99 ± 0.10	0.73 ± 0.09	1.09 ± 0.13	**0.02**
Caffeinate coffee intake (cup/day)	0.95 ± 0.10	0.70 ± 0.09	1.04 ± 0.13	**0.03**
Decaffeinated coffee intake (cup/day)	0.04 ± 0.01	0.03 ± 0.02	0.05 ± 0.02	0.44
Sweeten coffee intake (cup/day)	0.04 ± 0.01	0.03 ± 0.02	0.05 ± 0.02	0.41

A linear regression model was used to determine the *p*-value for continuous variables, which are presented as Mean ± SD.

Categorical variables are shown as percentages, and the chi-square test was used to determine the *p*-value. *p*-value ≤ 0.05 are highlighted in bold.

### Association between coffee, caffeine intake, and risk of PPD

The association between generic coffee, decaffeinated coffee, and caffeinated coffee and the risk of PPD is shown in Table 2.

**TABLE 2:** Generic coffee, caffeinated coffee, and decaffeinated coffee intake (cup/day) and the risk of PPD.

	Model 1	Model 2	Model 3
	**Odds ratio (95% CI)**	**Odds ratio (95% CI)**	**Odds ratio (95% CI)**
Generic coffee	**0.882(0.786,0.989)** *	0.904(0.815,1.002)	**0.889(0.802,0.985)** *
Decaffeinated coffee	0.800(0.430,1.488)	0.835(0.456,1.529)	1.043(0.586,1.856)
Caffeinated coffee	**0.888(0.794,0.993)** *	0.910(0.822,1.006)	**0.887(0.801,0.982)** *

Model 1 (crude model) did not adjust any factors, Model 2 adjusted for age and racial variables, and Model 3 adjusted for all variables. *p*-value ≤ 0.05 are highlighted in bold.

*p < 0.05.

Three weighted logistic regression models were established. Model 1 was established without adjustments (crude model). The results indicated that each increment of one cup in generic coffee and caffeinated coffee consumption was associated with an OR (95%) of 0.882 (0.786, 0.989) and 0.888 (0.794, 0.993), respectively, indicating a reduced risk of PPD of 11.2 and 11.1%, respectively. The trend in decaffeinated coffee consumption was not statistically significant. Model 2 was adjusted for age and race; however, the results were not statistically significant. Model 3 included all the relative covariates: age, BMI, race, educational level, ratio of family income to poverty, marital status, moderate activity status, breastfeeding status, postpartum time, smoking status, and insurance. The ORs (95% CIs) for per-cup increments of daily intake of generic coffee and caffeinated coffee were associated with a decrease in the risk of PPD of 11.1% (0.15–19.8%) and 11.3% (0.18–19.9%), respectively.

### Stratified analysis

Based on the above analysis, we speculated that there may be a negative association between coffee consumption and PPD. To further explore and verify the factors that affect this association, we performed a stratified analysis by race, breastfeeding status, and postpartum period, and quantified coffee consumption at three levels. Table 3 presents the subgroup analyses of the results of the fully adjusted model (Model 3). Overall, the group that consumed more than three cups of caffeinated coffee daily had a declining trend in PPD risk compared with the group that consumed less than one cup per day. In non-Hispanic white people, the ORs (95% CIs) of PPD for the group drinking three cups of caffeinated coffee per day against the group consuming less than one cup per day was 0.151 (0.038, 0.595) with a *p* < 0.001 significance level, which indicated a reduced PPD risk of 84.9%. In terms of breastfeeding status, women who were not breastfeeding benefitted from drinking more than three cups of caffeinated coffee per day with ORs (95% CIs) of 0.299 (0.117, 0.766) compared with mothers who drank less than one cup per day, with a significant difference. Women who were breastfeeding also had a reduced risk of PPD when drinking more than three cups of caffeinated coffee per day, although no statistical significance was observed.

**TABLE 3:** Association between caffeinated coffee intake (cup/day) and PPD stratified by groups.

	OR (95% CI)			
	**Less than 1 cup per day**	**1–3 Cups per day**	**≥3 Cups per day**	***p* for interaction**
Race/ethnicity				0.118
Other	ref	1.176(0.479,2.887)	0.883(0.144,5.404)	
Mexican American	ref	0.889(0.369, 2.143)	3.021(0.384,23.803)	
Non-Hispanic White	ref	1.144(0.572,2.291)	0.151(0.038,0.595)**	
Non-Hispanic Black	ref	0.331(0.061, 1.789)	0.707(0.040,12.569)	
Breastfeeding status				0.787
No	ref	0.976(0.606,1.572)	0.299(0.117,0.766)*	
Yes	ref	1.173(0.447,3.078)	0.519(0.129,2.092)	
Postpartum period				0.121
Within 1 year	ref	2.832(0.876,9.162)	0.444(0.042,4.672)	
1–2 years	ref	0.735(0.408,1.325)	0.145(0.036,0.584)**	
More than 2 years	ref	1.083(0.496,2.365)	0.793(0.204,3.081)	

*p < 0.05, **p < 0.01.

Previous research has indicated that the pathophysiology and pathogenesis of PPD may vary during various postpartum periods (Batt et al., 2020). In this study, the postpartum period was stratified as 1 year, 1–2 years, and more than 2 years. In the group of 1–2 years, the ORs (95% CIs) of PPD in the population drinking three cups of caffeinated coffee per day versus the group consuming less than one cup per day were 0.145 (0.036, 0.584) with a *p* < 0.001 significance level. Although there was a negative association between drinking more than three cups of caffeinated coffee and PPD in other time period subgroups (within 1 year or more than 2 years), no statistical difference was found. In addition, the interaction effect was evaluated to clarify the potential association between the stratifying variables and coffee consumption, but no statistically meaningful interaction effect was found.

## Discussion

The popularity of coffee and coffee beverages is increasing worldwide, and their effects on human physical and mental health have been extensively studied. Although numerous studies have demonstrated a potential negative association between coffee consumption and depression (Grosso et al., 2016; Navarro et al., 2018; Wang et al., 2016), evidence on postpartum women is sparse. PPD is often unrecognized and left untreated and it can have a profound impact on children’s parenting and development (Goodman, 2004; Psychosocial Paediatrics Committee, 2004; Ko et al., 2017). Therefore, we conducted this exhaustive, representative, cross-sectional study using the NHANES database from 2007 to 2018 and analyzed a representative sample of 821 postpartum women.

We found a negative independent association between coffee intake and postpartum depression. The results of the cross-study indicated that this negative correlation is statistically significant in generic and caffeinated coffee consumption, but not in decaffeinated coffee consumption. This suggests that the protective effect of coffee against PPD is most likely mediated by caffeine. Correspondingly, this beneficial association was found to be significant in multiple subgroups, including white individuals and women who were not breastfeeding. We concluded that women who are 1–2 years postpartum are more likely to benefit from this protective effect of coffee.

The main finding of this study is consistent with the conclusions of previous studies (Lucas et al., 2011; Lahortiga-Ramos et al., 2018; Kimura et al., 2020; Ma et al., 2022; Wang et al., 2023). In a 10-year follow-up study that included 14,413 male and female participants with a mean age of 36 years, the hazard ratio of depression in participants who consumed ≥ 4 cups of coffee daily was 0.37 compared with participants who drank less than one cup of coffee daily (Navarro et al., 2019). For older women, Lucas et al. (2011) established a prospective United States cohort of 50,739 women with a mean age of 63. After a 10-year follow-up, they found that consuming two to three cups or ≥4 cups per day could lower the risk of depression by approximately 10 and 15%, respectively. A similar effect was also observed in older Asian women (Kimura et al., 2020) with a more marked impact. In a recent meta-analysis of 11 publications and 330,677 individuals, a linear association between coffee consumption and depression was observed, with a decline of 8% in depression for each increment of one cup of coffee (Wang et al., 2016). Current studies have also provided some insights into the molecular interactions between caffeine and human neuro-activities (Fang et al., 2021).

Despite extensive evidence supporting the antidepressant benefits of coffee and caffeine, evidence involving postpartum women is still lacking. Possible reasons may include ethical and legal issues related to postpartum mental disorder screening (Gilbert et al., 2017; Vemuri, 2012), sparse demographic distribution, and low self-reporting rates (Simmons et al., 2007). Moreover, evidence recommending caffeine consumption guidelines for postpartum women is scarce, of poor quality, and equivocal (McCreedy et al., 2018). To the best of our knowledge, this is the first study to investigate the associations between coffee consumption, caffeine intake, and depression in postpartum women. The first benefit of our study is that we used 821 samples from the NHANES between 2007 and 2018, which is representative of 5.86 million United States residents, and offered a strong and trustworthy result with minimized sample bias. Second, we chose covariables based on the characteristics of the postpartum population and adjusted them based on outcome- and exposure-related factors, which strengthened the generalizability of the results. The findings of this study may therefore provide practical and instructional evidence for future research on the prevention of postpartum depression.

This study has several limitations that need to be clarified. First, this was a cross-sectional study, and the data on coffee drinking were obtained from 24-h dietary recall in MEC, which cannot reflect the dynamic changes in diet before and after pregnancy. Second, this study was unable to incorporate individual’s prepartum mental disorder and family history as covariables due to the inaccessibility of complete medical history. Last, although the results indicate that consuming three or more cups of caffeinated coffee per day can reduce risk of PPD, it is notable that no statistically significant results were found in the currently breastfeeding population. Thus, the results of this study should be interpreted with caution and should not be regarded as a recommendation for increased coffee consumption in whole-population during lactation.

As a result of the COVID-19 pandemic, the global incidence of PPD has climbed from 13 to 19% to 23 to 27% (Nisar et al., 2020). This dismal trend prompts more focus on the prevention and treatment of PPD. In addition to genetic and social variables, lifestyle factors, such as diverse diets that may contain moderate psychoactive substances, may have an impact on PPD (Gow et al., 2022). The discovery of these associations and the recommendation of a specific diet for postpartum populations will contribute to the reduction of PPD risk. Consequently, based on the findings of this study, we recommend that in future questionnaire surveys and cohort studies of postpartum populations, appropriate consideration should be given to enhancing the screening of dietary characteristics and evaluating their impact on the pathogenesis and treatment of PPD.

In conclusion, the results suggest that generic and caffeinated coffee consumption could have protective effects in PPD, and that consuming more than three cups of caffeinated coffee could lower risk of PPD. The subgroup analyses indicate that this tendency is more prominent in white people, women 1–2 years postpartum, and non-breastfeeding groups with statistical significance. A protective effect was not observed in decaffeinated coffee.

## Data availability statement

Publicly available datasets were analyzed in this study. This data can be found here: https://www.cdc.gov/nchs/nhanes/index.htm.

## Ethics statement

The studies involving human participants were reviewed and approved by NCHS Research Ethics Review Board. The patients/participants provided their written informed consent to participate in this study.

## Author contributions

YW collected the data and drafted the manuscript. ZW revised the manuscript. YX designed the study. BZ and PG analyzed the data. All authors contributed to the article and approved the submitted version.

## Footnotes

1
http://www.r-project.org
